# Quantifying single nucleotide variant detection sensitivity in exome sequencing

**DOI:** 10.1186/1471-2105-14-195

**Published:** 2013-06-18

**Authors:** Alison M Meynert, Louise S Bicknell, Matthew E Hurles, Andrew P Jackson, Martin S Taylor

**Affiliations:** 1 MRC Human Genetics Unit, MRC Institute for Genetics and Molecular Medicine, University of Edinburgh, Western General Hospital, Crewe Road, Edinburgh, UK; 2 Wellcome Trust Sanger Institute, Wellcome Trust Genome Campus, Hinxton, Cambridge, UK

## Abstract

**Background:**

The targeted capture and sequencing of genomic regions has rapidly demonstrated its utility in genetic studies. Inherent in this technology is considerable heterogeneity of target coverage and this is expected to systematically impact our sensitivity to detect genuine polymorphisms. To fully interpret the polymorphisms identified in a genetic study it is often essential to both detect polymorphisms and to understand where and with what probability real polymorphisms may have been missed.

**Results:**

Using down-sampling of 30 deeply sequenced exomes and a set of gold-standard single nucleotide variant (SNV) genotype calls for each sample, we developed an empirical model relating the read depth at a polymorphic site to the probability of calling the correct genotype at that site. We find that measured sensitivity in SNV detection is substantially worse than that predicted from the naive expectation of sampling from a binomial. This calibrated model allows us to produce single nucleotide resolution SNV sensitivity estimates which can be merged to give summary sensitivity measures for any arbitrary partition of the target sequences (nucleotide, exon, gene, pathway, exome). These metrics are directly comparable between platforms and can be combined between samples to give “power estimates” for an entire study. We estimate a local read depth of 13X is required to detect the alleles and genotype of a heterozygous SNV 95% of the time, but only 3X for a homozygous SNV. At a mean on-target read depth of 20X, commonly used for rare disease exome sequencing studies, we predict 5–15% of heterozygous and 1–4% of homozygous SNVs in the targeted regions will be missed.

**Conclusions:**

Non-reference alleles in the heterozygote state have a high chance of being missed when commonly applied read coverage thresholds are used despite the widely held assumption that there is good polymorphism detection at these coverage levels. Such alleles are likely to be of functional importance in population based studies of rare diseases, somatic mutations in cancer and explaining the “missing heritability” of quantitative traits.

## Background

Targeted capture and sequencing of human exons (exome-seq) is an increasingly popular addition to genotyping microarrays and a lower cost alternative to whole genome sequencing for researchers investigating heritable traits [1]. The complete human protein-coding exome comprises only 1% of the human genome, and it is typically in what would be considered the easier fraction to sequence and align uniquely to a reference genome. Most of the causal disease variants identified to date have been in protein-coding exonic sequence [2], and there are well established experimental paradigms to explore the functional consequences of amino acid changing variants. As the approach is sequence based, there is the potential to ascertain all simple sequence variants within the targeted regions in each member of a study cohort.

Exome-seq has been successfully applied to identify causal variants in a number of Mendelian genetic disorders [3-5]. These study designs often require only tiny cohorts with little or no pedigree information, making many diseases tractable to genetic study for the first time. Exome-seq is also being used to study somatic mutations in cancers [6] and has been proposed as a method to study complex traits where the ability to detect rare variants along with new variant discovery make it an ideal complement to association studies [7,8]. Causal variants for complex diseases previously identified through microarray-based association studies have been shown to localize in or near exonic sequence [9], and human exomes contain an excess of rare non-synonymous coding variants that could explain some part of the missing heritability problem [10].

For all re-sequencing projects (where there is a closely related reference sequence that can be used for assembly, rather than requiring *de novo* sequence assembly), the total amount of usable sequence is considerably less than the amount that comes off the sequencing machines. Two standard analysis steps reduce the amount. First, reads that cannot be uniquely mapped to the genome cannot be used. A read may be unmappable because a) it contains too many sequencing errors, b) it is relatively error free but contains too many non-reference bases, c) it maps to an insertion, deletion, or structural variant, or d) it maps to multiple positions in the genome equally well. Second, duplicate reads are removed. Duplication is generally defined as having identical position in the genome, but may or may not include identical read sequence. Removing duplicate reads minimises issues with PCR amplification and improves the specificity of variant calling [11].

Exome sequencing suffers from the additional problem of off-target reads, those generated from the genomic sample but outside the regions targeted for capture [12]. These reads can certainly be used for polymorphism discovery, but they are unlikely to map to protein-coding sequence or even regulatory features. After applying all three of these reductions, the percentage of usable on-target reads can be as low as 35% [3,4]. Read mapping also demonstrates a bias towards the reference sequence: reads that contain too many alternate alleles align with lower scores to the reference genome than reads with only reference alleles [13]. As a consequence of heterogeneity in target capture efficiency, amplification and the mapping of reads to the reference genome, usable reads are unevenly distributed over the targeted regions.

There has rightly been a great deal of focus on accurately measuring the specificity of single nucleotide variant (SNV) calls in next-generation sequence data (e.g. [11]). But it is also hugely important for study design and the interpretation of results to understand the sensitivity attained. How many SNVs are likely to have been missed and where are they most likely to be located? Ajay and colleagues [14] have gone some way to addressing this problem by showing that at the commonly used threshold of 30X coverage, 30% of protein coding regions have insufficient read depth to confidently call genotypes. We set out to extend this observation to a wide range of read depths and compare the consistency of SNV calling sensitivity between genomic regions.

To measure the sensitivity of SNV detection we have applied down-sampling to deeply sequenced exomes and asked how well the down-sampled alignments perform at calling known SNVs that are both present in the full alignment and genotyped by the HapMap Phase III project [15]. This approach provides us with a gold-standard set of SNVs that can be generated for any deeply sequenced human target and allows cross-comparison of platforms and laboratories without the need to repeatedly and deeply sequence a known reference. With this gold-standard we have investigated the relationship of SNV detection sensitivity to both average coverage over the entire targeted exome and per-nucleotide coverage for each variant.

Calibrating SNV detection sensitivity with per-nucleotide coverage allows us to estimate how many genuine SNVs may have been missed in a specific targeted region and evaluate whether an exon or gene can be considered thoroughly screened for SNVs in an individual. It also provides the potential to correct the observed rate of polymorphism for biases in coverage between genomic regions. This is crucial for the robust application of population based measures of mutation, selection and demography [16] that are becoming increasingly important for the functional interpretation of non-coding regulatory sequences [17].

The relationship between mean on-target read depth and SNV sensitivity is important for study design where a researcher must generally decide *a priori* how to handle the trade-off between the number of individuals to study and the depth at which to sequence them. To explore this relationship we have collected exome sequence data, generated at unusually deep coverage, from separate laboratories and utilising four different capture technologies. Our principal aim here is not a comprehensive comparison of competing exome capture technologies, such studies have been published elsewhere [12,18-20], rather to understand real world variances in target capture efficiency and uniformity. In the case of rare genetic disorders, the focus is on how much sequencing is required to confidently identify the majority of variants in the targeted regions of an individual. We address the problem in the context of both autosomal dominant and recessive disorders, in which both heterozygous and homozygous mutations may be implicated. In this work, we asked what level of mean on-target read depth is required to accurately identify a given percentage of SNVs, where accuracy includes position, alleles, and genotype.

## Results and discussion

### Simulating shallower sequencing

Thirty capture-targeted and sequenced human exomes, encompassing two different laboratories and four different capture methods, were aligned to the reference genome using a standard protocol (see Methods, Additional file 1: Table S1a,b). Twelve of these exomes are part of an ongoing disease study, subsequently denoted HW samples and the remaining 18 have been previously published [1,5]. Of the previously published exomes, eight were from individuals genotyped in the HapMap Phase III project [15], (Additional file 1: Table S1a). The deep sequencing of these exomes enabled us to explore SNV detection sensitivity by random down-sampling of reads to simulate shallower sequencing. The down-sampled alignments were randomly generated from the original “full alignments” by including each read with a given probability (see Methods). As the exomes were captured using a mixture of technologies by different laboratories, we defined a common set of targets using CCDS [21].

### SNV detection sensitivity as a function of read depth at a polymorphic site

We called SNVs on all full and down-sampled alignments using the Genome Analysis Toolkit (GATK) UnifiedGenotyper tool [11]. SNV calls were considered “true” if they were represented (matching position and alleles) in the set of polymorphic sites genotyped by the HapMap Phase III project [15], located within the CCDS target regions, and found in the full alignment for a given exome. The cross-reference to known polymorphic sites allowed us to include all SNV calls with a reasonable assumption of accuracy that did not require filtering on the variant quality score or read depth. We validated our true set of SNV calls for the eight HapMap exomes against the genotype calls from the HapMap project [15] and found concordance of 99.7 ± 0.1% for heterozygous SNVs and 99.1 ± 0.4% for homozygous SNVs.

SNV calls from the down-sampled alignments were labelled “positive” if they met the same criteria as the “true” SNV calls in the full alignments. We classified positive SNV calls according to whether or not they were observed in the set of true SNV calls for the exome, and whether or not the genotype of the calls matched. Positive SNV calls observed in the true set with matching genotype were labelled true positives; those with mismatched genotype were labelled partial true positives. Partial true positives can be of two types. The more common heterozygous true–homozygous positive SNV calls occur when the down-sampled alignment has too few reference allele reads to call the position heterozygous. Homozygous true–heterozygous positive SNV calls are the opposite case: when the down-sampled alignment has sampled by chance enough reads with the reference allele to call the position heterozygous, but given all the reads at that position, the variant calling algorithm identifies these as sequencing errors.

**Figure 1:**
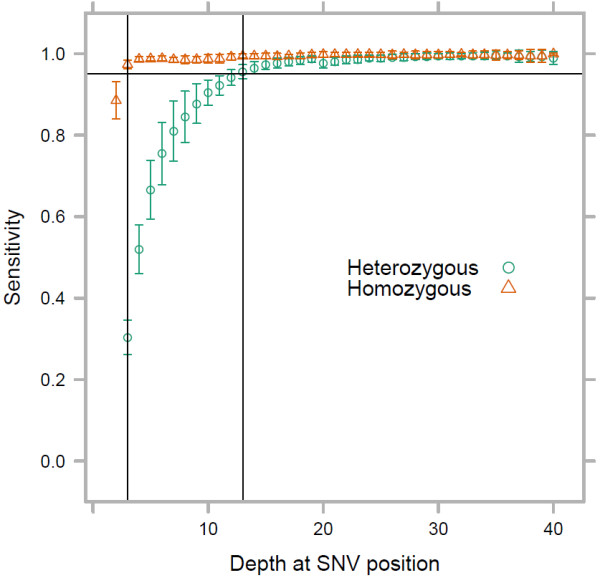
**SNV detection sensitivity as a function of depth at the polymorphic site.** Sensitivity of at least 95% is reached at depths of 3X for homozygous SNVs and 13X for heterozygous SNVs. Raw data for these curves is available (Additional file 2).

We measured sensitivity as a function of read depth at each true SNV site (Figure 1, Additional file 2) as described in Methods. Within the subset of CCDS targeted by all four capture methods, 95% detection sensitivity was reached for homozygous SNVs at depths of ≥ 3X and for heterozygous SNVs at depths of ≥ 13X. At positions with 10X read depth, only 90 ± 3% of heterozygous SNVs are correctly detected whereas the remaining 7–13% are typically called as non-polymorphic (Additional file 1: Table S2). At these lower read depths it was also common to observe partial true positive calls where heterozygous sites were miscalled as homozygous for the non-reference allele. Our results show that empirical sensitivity is substantially worse than that predicted from the naive expectation of sampling from a binomial (Additional file 1: Figure S2).

**Figure 2:**
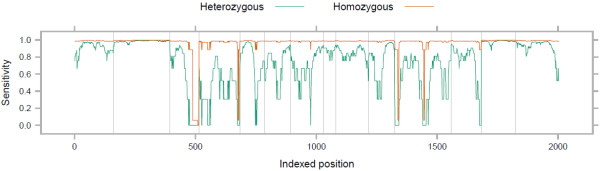
**SNV detection sensitivity across the example gene FERMT3 for the exome HW07.** Grey vertical lines denote exon boundaries.

Sensitivity in polymorphic sites used as our true set was higher than those identified in the full alignments within the shared CCDS targets and not occurring in the HapMap Phase III set (Additional file 1: Figure S1a). This is likely due to the higher rate of low quality variants in the latter set. The application of a commonly used Phred-scaled quality threshold of 30, corresponding to an expected 0.1% false call rate, to both the known and novel variants makes the two curves indistinguishable (not shown). The rate of missed heterozygote calls at low read depth is therefore not an unusual feature of sites genotyped by the HapMap project, and polymorphisms are generally called in the full alignments with high precision. No significant difference was observed between sensitivity of detecting homozygous SNVs in regions considered difficult (see Methods) to capture, sequence, and map, and SNVs in general; however, heterozygous SNVs in these regions were more easily detected at lower depths (Additional file 1: Figure S1b). This counterintuitive observation may relate to the typically higher G+C content of difficult regions (see below) and a consequent reduced bias for capturing the reference allele. The number of HapMap Phase III SNV sites in these difficult regions was relatively small (on average 283 ± 117 across the full CCDS target, compared to 1132 ± 419 for HapMap Phase III sites in the shared CCDS target), and none had read depth over 9X. There was no difference between the four capture methods in measured SNV detection sensitivity given the depth at a polymorphic site (Additional file 1: Figure S1c). Per-site on-target read depth is thus a good predictor of polymorphism detection sensitivity across target regions, between capture platforms and amongst laboratories. Using these results, sensitivity can be calculated across a gene or target set of interest to identify regions which are under-covered for the purpose of variant detection (e.g. Figure 2). Software to achieve this is provided (Additional file 3).

### SNV detection sensitivity as a function of mean on-target read depth

**Figure 3:**
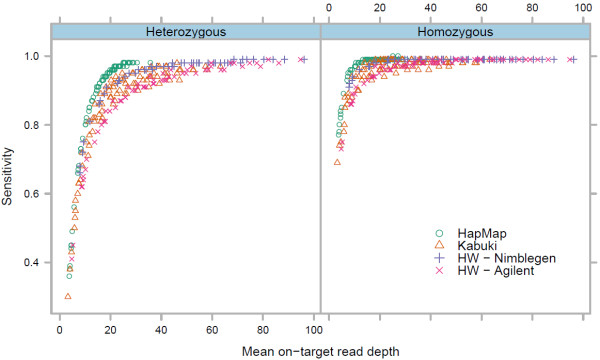
**SNV detection sensitivity as a function of mean on-target read depth.** Mean on-target read depth is calculated using bases aligned to the set of target regions covered by all exome capture platforms.

Using the sensitivity curves described in Figure 1, we calculated the total potential detection sensitivity of targeted regions for the 30 exomes. Because of differences in target probe design, we used the CCDS exon definitions (see Methods). Total detection sensitivity for an exome was defined as the sum across all positions in the target regions of the depth at that position multiplied by the mean SNV detection sensitivity for that depth, calculated separately for both heterozygous and homozygous SNVs (Figure 3). We estimated the mean on-target read depth required for achieving varying levels of total potential recall on the CCDS target set for the four capture methods separately and together (Table 1). Using the complete set of CCDS target regions highlights the differences in probe design between the four capture methods(Additional file 1: Figure S3a), while examining the subset of regions covered by at least one exome for each method individually (see Methods) gives a very similar result to the set of regions targeted by all four methods (Additional file 1: Figure S3b).

**Table 1:** Mean on-target read depth required for varying SNV detection sensitivity in targeted regions

	**Total detection sensitivity**											
	**80%**				**90%**				**95%**			
**Source**	**Het**		**Hom**		**Het**		**Hom**		**Het**		**Hom**	
HapMap	11.1 ±	0.9	5.3 ±	1.2	17.2 ±	0.9	8.3 ±	1.1	19.7 ±	0.9	9.0 ±	1.1
Kabuki	14.9 ±	1.9	7.6 ±	1.6	27.7 ±	6.1	12.2 ±	2.0	34.5 ±	8.4	18.0 ±	4.9
HW - Nimblegen	15.9 ±	2.5	9.3 ±	1.6	24.5 ±	1.9	9.3 ±	1.6	31.1 ±	2.7	15.9 ±	2.5
HW - Agilent	18.4 ±	1.6	9.5 ±	0.8	36.5 ±	3.5	15.2 ±	2.8	45.6 ±	4.2	21.7 ±	2.8

HW = Human Genetics Unit/Wellcome Trust Sanger Institute.

Different capture technologies have varying levels of target uniformity and efficiency. Exomes captured by the two custom array-based methods (HapMap and Kabuki) had the most uniform coverage, followed by the HW-Nimblegen exomes (Additional file 1: Figure S4). Other researchers have also observed higher uniformity of coverage in exomes captured by Nimblegen array kits compared to Agilent kits [18,19,22]. This difference is likely due in part to the different probe design strategies taken by Nimblegen and Agilent, as Nimblegen’s SeqCap EZ solution-based kit also generates more uniform coverage than Agilent kits [12]. The lower uniformity of read depth in the HW-Agilent exomes explains the higher levels of mean on-target read depth required to achieve similar levels of SNV detection sensitivity.

### Replicate samples

Two of the HW samples were captured once by each of the two capture methods (Agilent and Nimblegen). We compared the SNV calls in the subset of of CCDS targeted by all four capture methods from the full alignments of the replicates. For SNVs in the HapMap Phase III set where genotypes were called in both samples, concordance was >99% for both replicate pairs and for both heterozygous and homozygous genotypes. For novel sites, concordance was >98% for heterozygous sites and >95% for homozygous sites. The main difference between the replicates was in the number of novel SNVs called from the HW-Agilent samples that did not appear in the set of SNVs called from the HW-Nimblegen samples. 66% of novel SNVs called from HW10 (Agilent) were not called from HW03 (Nimblegen); likewise 54% from HW11 (Agilent) were not called from HW05 (Nimblegen). This was not due to lack of read depth in the Nimblegen samples at these sites (Additional file 1: Figure S5); in fact, the Nimblegen samples had greater depth at these sites on average (one-sided Wilcox rank sum test *p* < 2 × 10^−16^ for both replicate pairs). Sample HW07, which was captured and sequenced at the same time as samples HW10 and HW11, also has roughly 2.5X as many novel heterozygous variant calls as the other 25 samples (Additional file 1: Table S1c). This appears to be an experimental batch effect.

### Characteristics of “difficult” target regions

The target regions were split into maximum 100bp non-overlapping tiles and classified as “difficult” or “easy” to capture, sequence and map for each exome (see Methods). Consistent with earlier reports [3,12,20,23], tiles classified as difficult in at least five of the sequenced exomes had higher G+C content compared to those classified as easy in at least five (one-sided Wilcox rank sum test *p* < 2 × 10^−16^; Additional file 1: Figure S6). Difficult tiles were more likely to be annotated with simple repeats than easy tiles (*χ*^2^ test *p* < 2 × 10^−16^). Bainbridge and colleagues also noted that low complexity regions were less likely to be well-covered [23]. This is likely due to a combination of the difficulty in uniquely mapping reads to these regions, and in designing probes for them. Across all 30 exomes, on average 63 ± 21% of difficult target region tiles were shared between any given pair of exomes, implicating technology-agnostic issues such as read mappability. There was more variation among easy target region tiles, with only 36 ± 22% of such tiles shared on average across all exomes. However, exomes captured by the same or similar method were more likely to have difficulty with certain target tiles (Additional file 1: Figure S7).

## Conclusions

We used down-sampling of deeply sequenced exomes along with cross-referencing to known SNV sites to measure how read depth influences SNV detection sensitivity. The considerable per-nucleotide sequence depth required to achieve 95% sensitivity for heterozygous SNVs highlights a substantial missing-data problem that is often overlooked in next-generation sequence analysis. For example, Ng and colleagues [5] used 8X coverage of a site as a threshold to call polymorphisms, but at this coverage we find that 16% of heterozygous polymorphisms are missed by the standard genotype calling strategies (Figure 1, Additional file 1: Table S2). Raising the coverage threshold to 10X (10% missing heterozygote calls) reduces the expected number of missing variant calls, but they remain substantial.

The problem of missing heterozygote SNVs stems from the stochastic and possibly also biased sampling of alleles by sequence reads [20]. If the reference genome allele is more sampled, non-reference alleles may be either not represented or at an insufficient frequency to produce a confident (exceeding a quality score threshold) genotype call. In contrast, if the non-reference allele is more abundantly sampled, information in the reference sequence is used to bolster the confidence of a reference allele call. Consequently the missing variant calls are specifically biased to non-reference alleles in the heterozygous state, precisely the alleles that are most likely to be of interest in population based (non-consanguineous) studies of rare disease and somatic cancer mutations.

We are not advocating the use of an excessively deep threshold to call polymorphisms; it makes sense to maximally use the available sequence information in an attempt to call variants even in regions of low sequence coverage. However it is important to quantify how likely a polymorphism is to remain undetected. This quantification can be achieved with the down-sampled alignment based calibrated data (Figure 1, Additional file 1: Table S2) that we have shown is portable between laboratories, platforms and agnostic to sequence quality score filtering. This will allow genes of interest to be scored according how thoroughly they have been screened for variants and identify sub-regions that may warrant follow-up targeted sequencing to improve coverage.

We have demonstrated that mean on-target read depth of 17–37X is required to identify 90% of heterozygous SNVs in the targeted regions for a given capture method, depending primarily on uniformity of read coverage. Heterogeneity in read coverage is inherent in all sequencing technologies but it is particularly pronounced in target capture approaches. This unevenness of coverage is the main reason it requires every target nucleotide to be sequenced dozens of times, to ensure sufficient coverage over the difficult targets to achieve reasonable sensitivity. Some exons are persistently difficult to capture for multiple methods. Our sensitivity estimates are based on the set of SNVs that can be discovered in the alignments with the maximum available data, so they naturally exclude some real SNVs in these difficult target regions. It is possible that a second round of capture, focusing on difficult exons, such as that carried out by Ng and colleagues [1], may be necessary in rare disease projects if no probable candidates are found.

Rates of polymorphism and allele frequencies within regions of the genome or categories of annotated sites can be used to help measure aspects of demography, selection and mutation [16]. But if there is a systematic bias in polymorphism detection sensitivity between regions of a genome being compared (for example a category of sequence biased towards difficult to capture sequence), these important measures will be distorted and undermined. Again using the single nucleotide resolution calibrated data (Additional file 1: Table S2) the average variant detection sensitivity of genomic regions or categories of sites could be compared and if necessary a correction applied in the case that sensitivity is not balanced between comparators.

Despite the missing polymorphism problem, from an analysis perspective the most immediate challenge is more likely to be too many probable candidates, and prioritisation of candidate causal variations and genes will be required. The rare genetic disorders that have been investigated using exome-seq so far have proved amenable to stringent filtering of known variation followed by damage prediction. As technology advances and sequencing becomes cheaper, researchers will need new methods to handle larger data sets and the inevitable inclusion of previously observed variants as disease or trait contributing candidates.

With rapid advances in sequencing technology there will soon come a cross-over point where whole genome sequencing is cheaper than exome-seq. The methods and missing variant-call problem described here apply not only to exome-seq but also to whole genome sequencing. The capturing of smaller target sets but across many more patients, for example to validate a substantial genetic effect contributed by multiple rare variants at a locus [24], is likely to be common in the future. Estimating the likelihood of missing heterozygous SNVs in a sequenced sample will be an integral part of analyzing the results of all such studies.

## Methods

### Exome capture and sequencing

Six of the HW exomes were captured using a Roche NimbleGen 2.1M array kit targeting exonic sequence as described by CCDS [21], and the other six with the Agilent SureSelect All Exon 38M solution kit targeting exonic sequence annotated by the GENCODE Consortium [25]. Paired-end reads of 54 bp were generated on the Illumina GAII platform.

Two individuals were captured twice, once with each technology. Exomes HW03 and HW10 were sampled from the same individual; likewise exomes HW05 and HW11.

FASTQ reads for 8 HapMap exomes [1] were obtained from the NCBI Sequence Read Archive [26]. These exomes were captured with two custom Agilent 244K microarrays and single-end 76 bp reads generated on the Illumina GAII platform. FASTQ reads for 10 Kabuki case exomes [5] were obtained from the NCBI dbGAP resource [27]. These exomes were captured on a custom Agilent 1M aCGH array and a mix of single- and paired-end 76 bp reads generated on the Illumina GAII platform. See Additional file 1: Table S1 for accessions. Additional file 1: Table S3 summarises sequence and capture technology used to generate each of the four data sets: HapMap, Kabuki, HW-Nimblegen and HW-Agilent.

### Ethical approval and consent

Informed consent was obtained from the families of all patients involved in the study HW data set. The study was approved by the Multi-centre Research Ethics Committee for Scotland (04:MRE00/19).

### Alignments

Reads for all 30 exomes were aligned to the hg19/GRCh37 assembly of the human genome reference sequence with BWA 0.5.9 [28]. Duplicate reads were removed using the MarkDuplicates function of Picard 1.79 (http://picard.sourceforge.net). Reads were re-aligned around indels and quality scores re-calibrated using the Genome Analysis Toolkit (GATK), release 2.2–8-gec077cd [29]. Full parameters are given in the Additional file 1. We randomly down-sampled reads from exome alignments using Picard DownsampleSam, which maintains read pair information. The probability of sampling each read varied from 0.1 to 0.9 at intervals of 0.1.

### Target regions

We defined a set of target regions using the 25 October 2012 CCDS exon definitions [21]. Overlapping coordinates from CCDS genes with status “Public” were merged so that every position was represented only once. Read depth at a particular base in a target region was defined as the total number of reads overlapping the position. This included reads which aligned with a gap to the position. The target regions were split into maximally 100 bp non-overlapping tiles for further analysis, with small tiles at target region edges. We defined the set of targeted regions for each capture method as the set of tiles which were covered by at least one read in at least one exome for that method. The intersection of the four target sets thus defined was used to compare sensitivity across capture methods.

We considered a base well-covered if it had a read depth of at least 10, and a target region tile as well-covered if at least 90% of its bases were well-covered. “Difficult” target region tiles had no well-covered bases in the full alignments. “Easy” target regions were well-covered in the downsampled alignments with read inclusion probability 0.1.

SRS086455 from the Kabuki exomes and HW06 from the HW-Nimblegen exomes each had considerably fewer well-covered targets than the other exomes from their respective sources, indicating partial capture failure. These two samples were excluded from all summary statistics and figures.

### Variants

Variants were called on the full and down-sampled alignments using Samtools 0.1.12a [30]. An independent set of variant calls was made using the Genome Analysis Toolkit (GATK) UnifiedGenotyper tool, release 2.2–8-gec077cd [11] (full parameters in Additional file 1). We obtained HapMap Phase III sites and genotypes from the project FTP site [15]. Variants from this set were mapped by position and alleles to called variants in the full and down-sampled alignments. Genotypes were mapped for the 8 HapMap exomes.

### Sensitivity by depth at position

Sensitivity for a given genotype *g* (heterozygous or homozygous) and read depth *d* was calculated as:

$$\frac{\text{TP}}{\text{TP}+\text{PTP}+\text{FN}}$$

where TP and PTP were the number of correctly positioned SNV calls of genotype *g* at read depth ≤ *d* with correct or incorrect genotype respectively and FN was the number of SNV calls of genotype *g* made in the full alignment where there was no corresponding call made in any down-sampled alignments with read depth ≤ *d* at that position. See Additional file 1 for a worked example.

## Availability of supporting data

Reads for the HW exomes are available upon request.

## Competing interests

The authors declare no competing financial interests.

## Authors’ contributions

AM and MT designed the analyses and wrote the manuscript. MT conceived of the study and AM performed the analysis. AJ, LB and MH provided and sequenced the 12 HW exomes. All authors read and approved the final manuscript.

## Supplementary Material

Additional file 1 **Supplementary information.** Supplementary information, figures, and tables. Click here for file

Additional file 2 **recall.tsv.** Tab-delimited text file containing empirical sensitivity estimates for read depths up to 100X. Click here for file

Additional file 3 **depth_and_recall.r.** R scripts for applying empirical sensitivity curve to exome alignment data. Click here for file
