# Supplementary material for: Quantifying single nucleotide variant detection sensitivity in exome sequencing
Source: BMC Bioinformatics. 2013 Jun 18;14:195. doi: 10.1186/1471-2105-14-195 (PMC3695811; doi:10.1186/1471-2105-14-195)
Supplement: Additional file 1 — Supplementary information. Supplementary information, figures, and tables. [file 1471-2105-14-195-S1.pdf]

Software commands and parameters

Extraction of FASTQ

From Sequence Read Archive (SRA) format files (HapMap and Kabuki exomes). Fastq-dump is available from http://trace.ncbi.nlm.nih.gov/Traces/sra/sra.cgi?view=software.

```
fastq-dump -A SRRxxxxxx SRRxxxxxx
```

Alignment of single-end runs with BWA

```
bwa aln -q 30 hg19.fasta sample_run.fastq > sample_run.sai
bwa samse hg19.fasta sample_run.sai sample_run.fastq
    | gzip > sample_run.bwa.sam.gz
```

Alignment of paired-end runs with BWA

```
bwa aln -q 30 hg19.fasta sample_run_1.fastq > sample_run_1.sai
bwa aln -q 30 hg19.fasta sample_run_2.fastq > sample_run_2.sai
bwa sampe hg19.fasta sample_run_1.sai sample_run_2.sai
    sample_run_1.fastq sample_run_2.fastq | gzip > sample_run.bwa.sam.gz
```

Further alignment with Stampy, keeping well-aligned reads from BWA

Stampy is available from http://www.well.ox.ac.uk/project-stampy.

```
samtools view -hbS -o sample_run.bwa.bam sample_run.bwa.sam.gz
python stampy.py --keepreforder -g hg19 -h hg19
    --bamkeepgoodreads -M sample_run.bwa.bam | gzip -c > sample_run.sam.gz
samtools view -hbS -o sample_run.unsorted.bam sample_run.sam.gz
samtools sort sample_run.unsorted.bam sample_run
```

Merge multiple runs

```
for each run:
    samtools view -H sample_run_X.bam | grep ^\@RG > sample_run_X.header.rg
    samtools view -H sample_run_X.bam | grep -v ^\@RG > sample.header
cat sample.header sample_run_1.header.rg ... sample_run_N.header.rg > sample.header.txt
samtools merge -h sample.header.txt sample.pre.rmdup.bam sample_run_1.bam ...
    sample_run_N.bam
```

Refine BAM file

Remove duplicates, re-align around indels, re-calibrate quality scores

```
java -Xmx4g -jar MarkDuplicates.jar
    INPUT=sample.pre.rmdup.bam
    OUTPUT=sample.bam
    METRICS_FILE=sample.rmdup.metrics.txt
    ASSUME_SORTED=TRUE
    VALIDATION_STRINGENCY=SILENT

java -Xmx16g -jar GenomeAnalysisTK.jar
    -T RealignerTargetCreator
```

```
    -R hg19.fasta
    -known 1000G_phase1.indels.hg19.vcf
    -known Mills_and_1000G_gold_standard.indels.hg19.vcf
    -o sample.intervals
    -I sample.bam

java -Xmx16g -jar GenomeAnalysisTK.jar
    -T IndelRealigner
    -R hg19.fasta
    -known 1000G_phase1.indels.hg19.vcf
    -known Mills_and_1000G_gold_standard.indels.hg19.vcf
    -targetIntervals sample.intervals
    -I sample.bam
    -o sample.realigned.bam

java -Xmx16g -jar GenomeAnalysisTK.jar
    -T BaseRecalibrator
    -R hg19.fasta
    -knownSites dbsnp_137.hg19.vcf
    -I sample.realigned.bam
    -o sample.recal.grp

java -Xmx16g -jar GenomeAnalysisTK.jar
    -T PrintReads
    -R hg19.fasta
    -BQSR sample.recal.grp
    -I sample.realigned.bam
    -o sample.recal.bam
```

Down-sampling BAM files

Duplicates are re-marked to handle cases where reads are no longer duplicates after down-sampling.

```
for p from 0.1 to 0.9:
    java -Xmx4g -jar DownsampleSam.jar
        INPUT=sample.recal.bam
        OUTPUT=sample.\$p.pre.rmdup.bam
        PROBABILITY=\$p
        R=null

    java -Xmx4g -jar MarkDuplicates.jar
        INPUT=sample.\$p.pre.rmdup.bam
        OUTPUT=sample.\$p.bam
        METRICS_FILE=sample.\$p.rmdup.metrics.txt
        ASSUME_SORTED=TRUE
        VALIDATION_STRINGENCY=SILENT
```

Coverage and statistics

CCDS genes were downloaded from ftp://ftp.ncbi.nlm.nih.gov/pub/CCDS/current_human/CCDS.20121025.txt on 21 March 2013. Including only rows with status “Public”, genes were split into protein-coding exonic regions and formatted into BED track format (http://genome.ucsc.edu/FAQ/FAQformat.html#format1).

Overlapping or abutting regions were merged to avoid double-counting. The merged regions were split into adjacent tiles of 100bp each.

```
java -Xmx4g -jar GenomeAnalysisTK.jar
    -T DepthOfCoverage
    -R hg19.fasta
    -I sample.bam
    -L CCDS.20121025.exons.tiled.bed
    -omitLocusTable
    -omitIntervals
    -o sample.depths.tsv

java -Xmx4g -jar GenomeAnalysisTK.jar
    -T CountBases
    -R hg19.fasta
    -I sample.bam

java -Xmx4g -jar GenomeAnalysisTK.jar
    -T CountReads
    -R hg19.fasta
    -L CCDS.20121025.exons.merged.bed
    -I sample.bam
```

Variant calling

```
java -Xmx4g -jar GenomeAnalysisTK.jar
    -T UnifiedGenotyper
    -R hg19.fasta
    -L CCDS.20121025.exons.merged.bed
    --dbsnp dbsnp_137.hg19.vcf
    -I sample.bam
    -o sample.vcf
    -stand_call_conf 30
    -stand_emit_conf 10
    -rf BadCigar
    -glm BOTH

map VCF to GRCh37 for snpEff annotation

java -jar snpEff.jar
    -c snpEff.config
    -o vcf
    -s sample.snpeff.html
    -no-intergenic
    -ud 1000
    GRCh37.68
    sample.grch37.vcf > sample.grch37.snpeff.vcf
```

Detection sensitivity calculation example

From Methods:

Sensitivity for a given genotype g (heterozygous or homozygous) and read depth d was calculated as:

$$\frac{TP}{TP + PTP + FN}$$

where TP and PTP were the number of correctly positioned SNV calls of genotype g at read depth $\leq d$ with correct or incorrect genotype respectively and FN was the number of SNV calls of genotype g made in the full alignment where there was no corresponding call made in any reduced alignments with read depth $\leq d$ at that position.

In this example, we consider a set of three sites which are heterozygous in an individual, and that all three are correctly identified in the full alignment of the exome captured for that individual. The reduced alignments produce the following data, where a genotype of 0/1 is heterozygous reference, 0/0 is homozygous reference, and 1/1 is homozygous non-reference:

Alignment size (G)	Site 1		Site 2		Site 3	
	Depth	Genotype	Depth	Genotype	Depth	Genotype
0.05	0	0/0	1	0/0	0	0/0
0.10	1	0/0	2	1/1	0	0/0
0.15	0	0/0	3	1/1	0	0/0
0.20	2	1/1	2	1/1	0	0/0
0.25	1	0/0	3	1/1	0	0/0
0.50	3	1/1	5	0/1	0	0/0
0.75	3	0/1	8	0/1	2	0/0
1.00	4	0/1	12	0/1	3	0/1

We sort each site's genotype calls by depth, and classify them as TP, PTP, or FN. If there are different genotype calls at the same depth, they are weighted by the number of occurrences. For example, at depth 3, site 1 is called once as homozygous and once as heterozygous. Therefore it contributes 0.5 to the PTP and 0.5 to the TP count at that depth. If there is no data for a given depth, the genotype calls from that depth minus one are used, recursively.

Depth	Genotypes			Classifications			
	Site 1	Site 2	Site 3	TP	PTP	FN	Sensitivity
0	0/0 (2)		0/0 (6)	0	0	2	0
1	0/0 (2)	0/0 (1)		0	0	3	0
2	1/1 (1)	1/1 (2)	0/0 (1)	0	2	1	0
3	0/1 (1), 1/1 (1)	1/1 (2)	0/1 (1)	1.5	1.5	0	0.5
4	0/1 (1)			2	1	0	0.67
5		0/1 (1)		3	0	0	1
6				3	0	0	1
7				3	0	0	1
8		0/1 (1)		3	0	0	1
9				3	0	0	1
10				3	0	0	1
11				3	0	0	1
12		0/1 (1)		3	0	0	1

Applying the sensitivity calibration curve

We provide the data for the sensitivity calibration curve in the file `recall.tsv`. This can be used to calculate and plot sensitivity across a given genomic region or set of regions, as in Figure 3. It can also be used to summarize the total detection sensitivity for a genomic region or set of regions, as we demonstrated for the targeted exome in Figure 2 and Supplementary Figure 3. This is done using the Genome Analysis Toolkit (McKenna *et al.*, 2010) and a custom R script.

Calculate and plot sensitivity across a genomic region

The coordinates of a genomic region in 1-based, end-inclusive format are in a tab-delimited text file called `regions.bed`. In this example, the coordinates are for the exons of FERMT3, the gene shown in Figure 3.

```
chr11 63974837 63974996
chr11 63978083 63978316
chr11 63978524 63978643
chr11 63978747 63978915
chr11 63979117 63979219
chr11 63986723 63986830
chr11 63986996 63987130
chr11 63987212 63987261
chr11 63987351 63987487
chr11 63987692 63987798
chr11 63987908 63988141
chr11 63988488 63988612
chr11 63990520 63990661
chr11 63990785 63990964
```

Coverage across the region for a given exome alignment is calculated using the GATK DepthOfCoverage tool. The tool will generate a tab-delimited text file containing the read depth at every position in the given region.

```
java -jar GenomeAnalysisTK.jar
    -T DepthOfCoverage
    -R hg19.fasta
    -I exome.bam
    -L regions.bed
    -omitSampleSummary
    -omitIntervals
    -o depths.tsv
```

In R, this can be plotted in combination with the sensitivity calibration information from the file `recall.tsv`.

```
source("depth_and_recall.r")
library(lattice)
depths = read.target.depths.and.recall("depths.tsv", "recall.tsv")
breaks = calculate.breaks(depths)
plot.recall.over.target(depths, breaks)
```

Summarize total detection sensitivity

For a summary statistic describing the quality of an entire exome capture or a particular subset of targets, we provide the total detection sensitivity for heterozygous and homozygous SNVs. This uses the `depths.sample_cumulative_coverage_counts` output from DepthOfCoverage.

```
source("depth_and_recall.r")
targets = read.table("targets.bed", col.names=c("chr", "start", "end")
target.length = sum(targets$end - targets$start)
recall = summarize.recall("depths.tsv.sample_cumulative_coverage_counts",
         "recall.tsv", target.length)
```

The data frame `recall` looks like this:

```
      genotype recall_low recall_mean recall_high
1 Heterozygous  0.7532072   0.7700903   0.7869733
2   Homozygous  0.8595090   0.8713913   0.8831860
```

To calculate the expected number of missed SNVs at a given mean on target read depth, as given in the abstract, we identified the downsampled alignment with mean depth closest to the target for each exome, in this case 20X. We then summarized the total detection sensitivity for each of the samples as above, and took the mean $\pm$ one standard deviation of estimated mean recall across the samples.

Supplementary Figure 1: SNV sensitivity as a function of depth at position

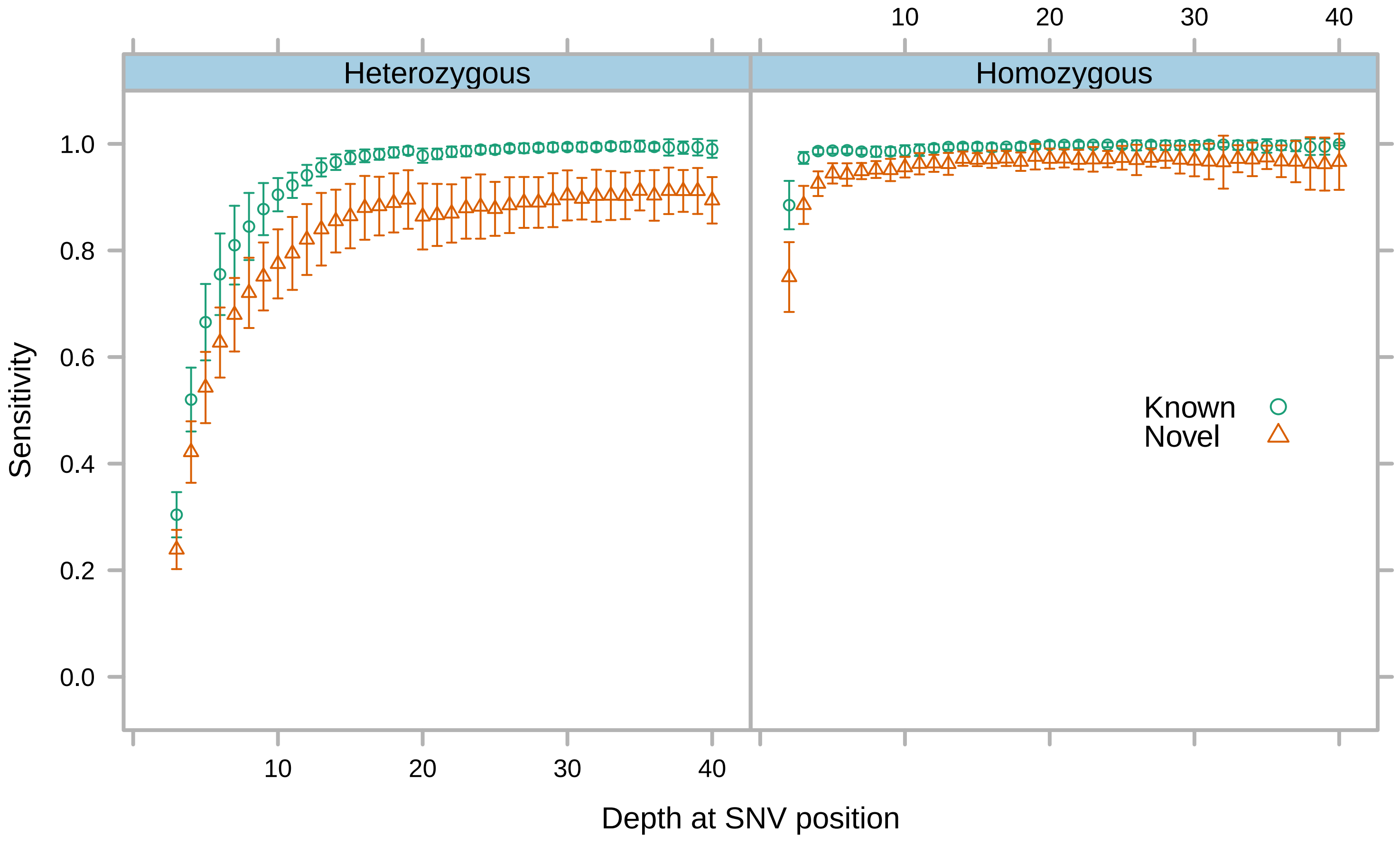

(a) Known (HapMap3) and novel SNVs. Novel SNV calls have no cross-reference to filter out false positives.

(b) Known SNVs in all tiles and difficult tiles.

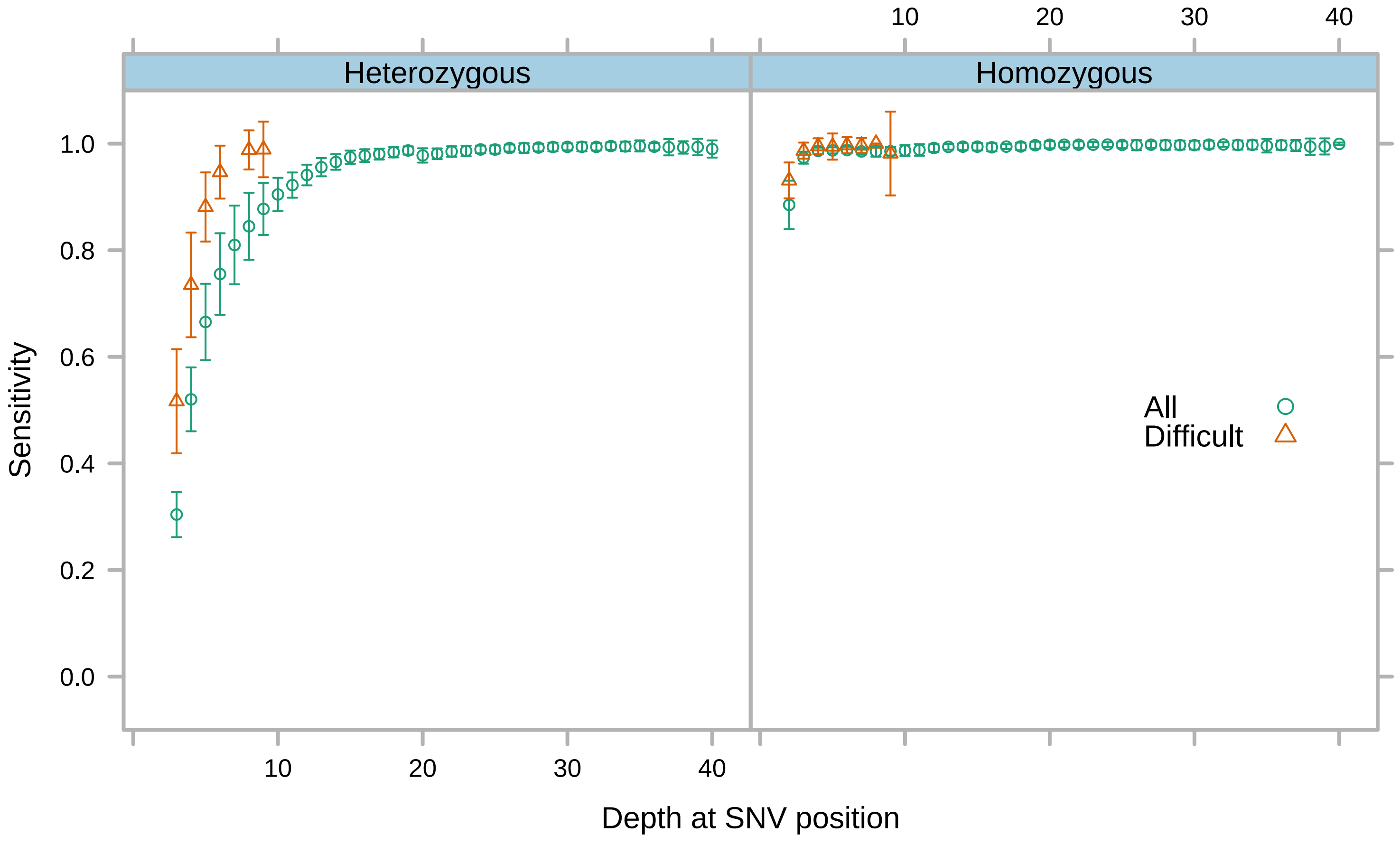

(c) Known SNVs by capture method.

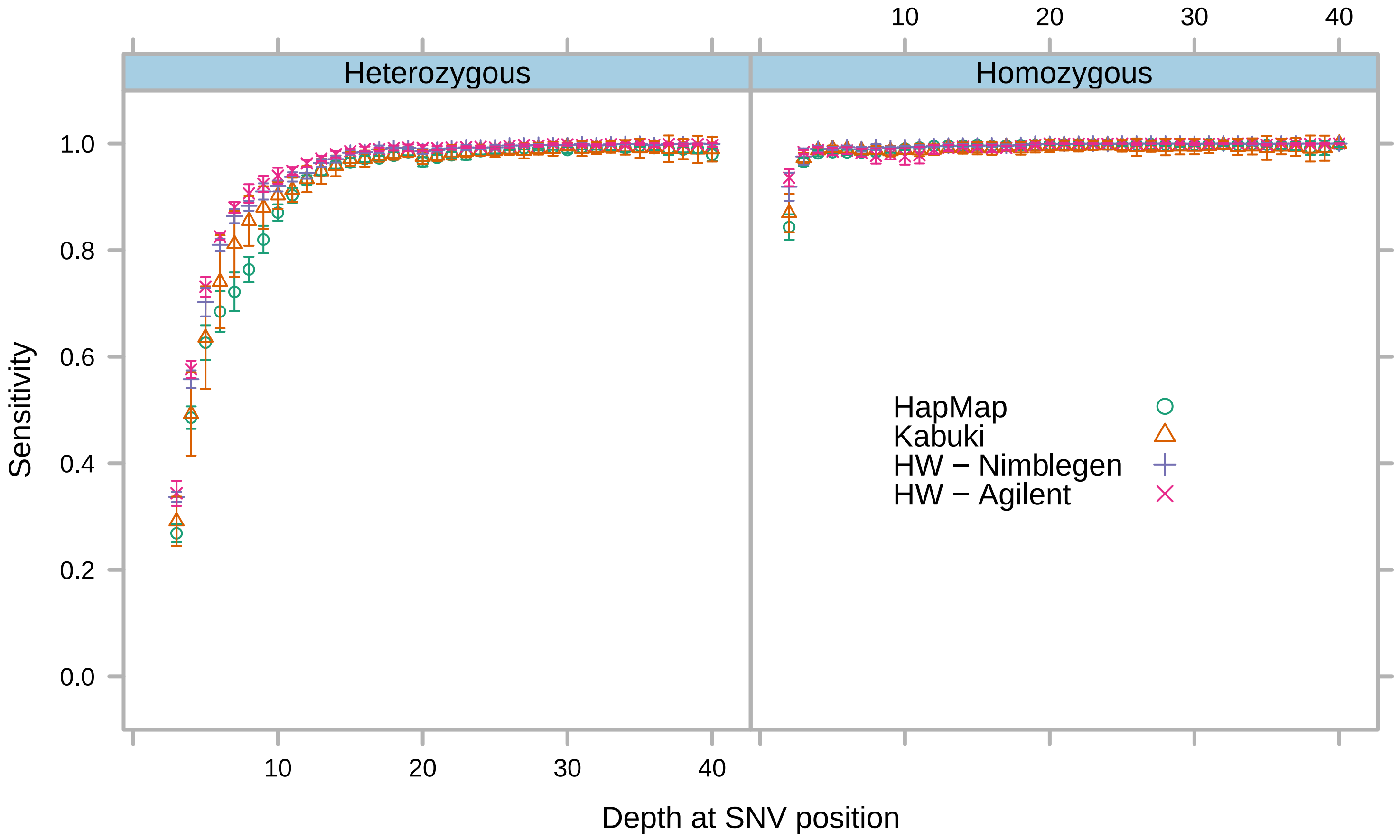

Supplementary Figure 2: Empirical heterozygous SNV sensitivity compared to theoretical heterozygous SNV sensitivity based on the binomial distribution as described in Chang *et al.* (2011).

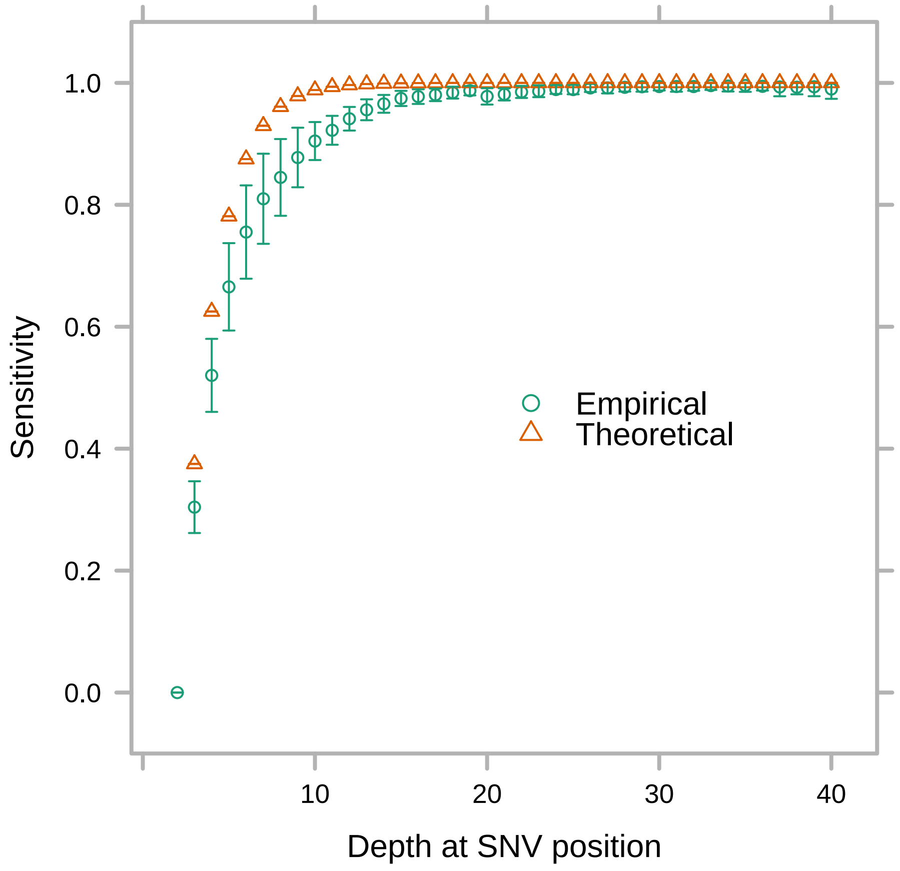

Supplementary Figure 3: SNV sensitivity against mean on-target read depth

(a) Complete CCDS target.

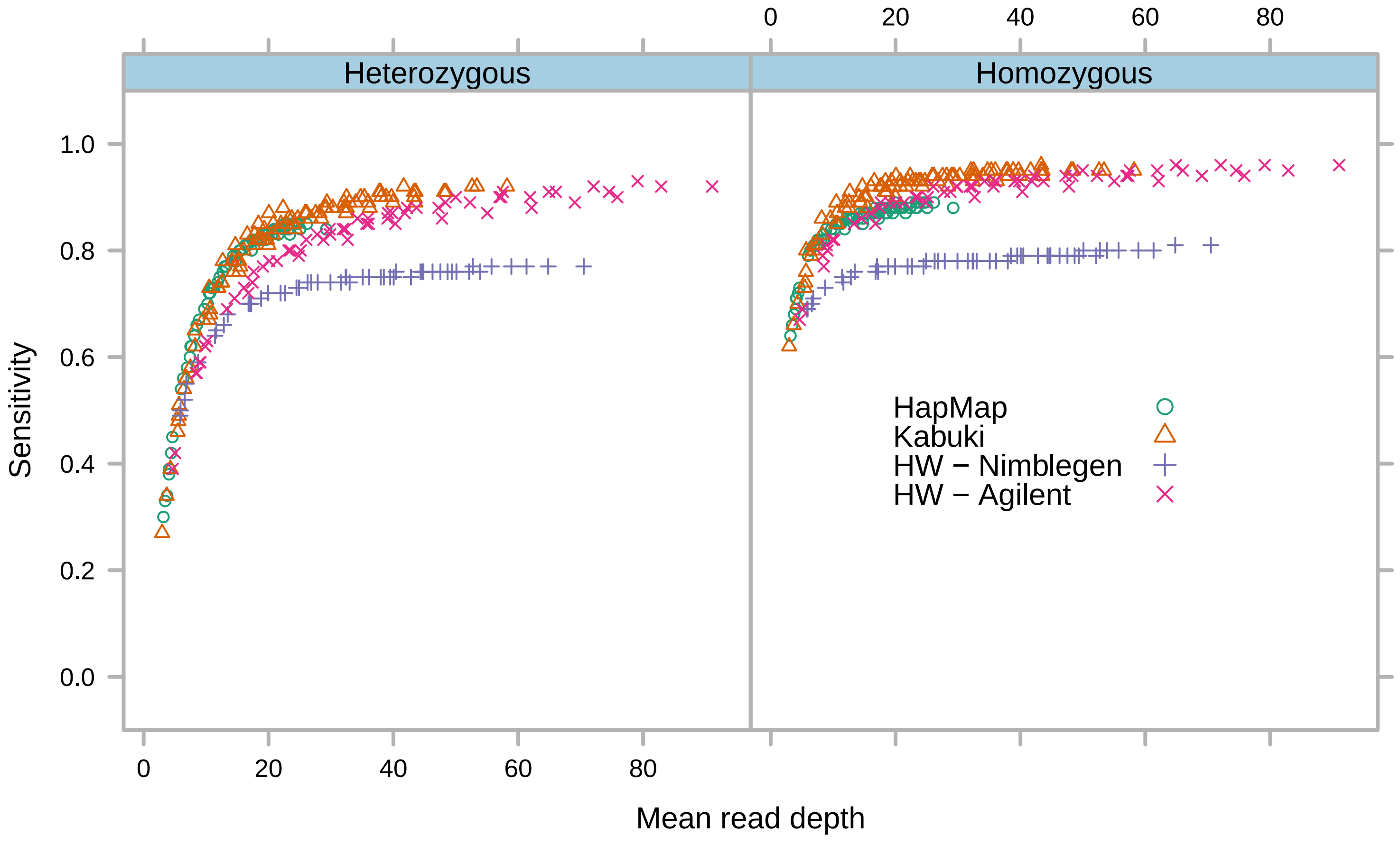

(b) Subset of CCDS targets covered by each exome capture method individually.

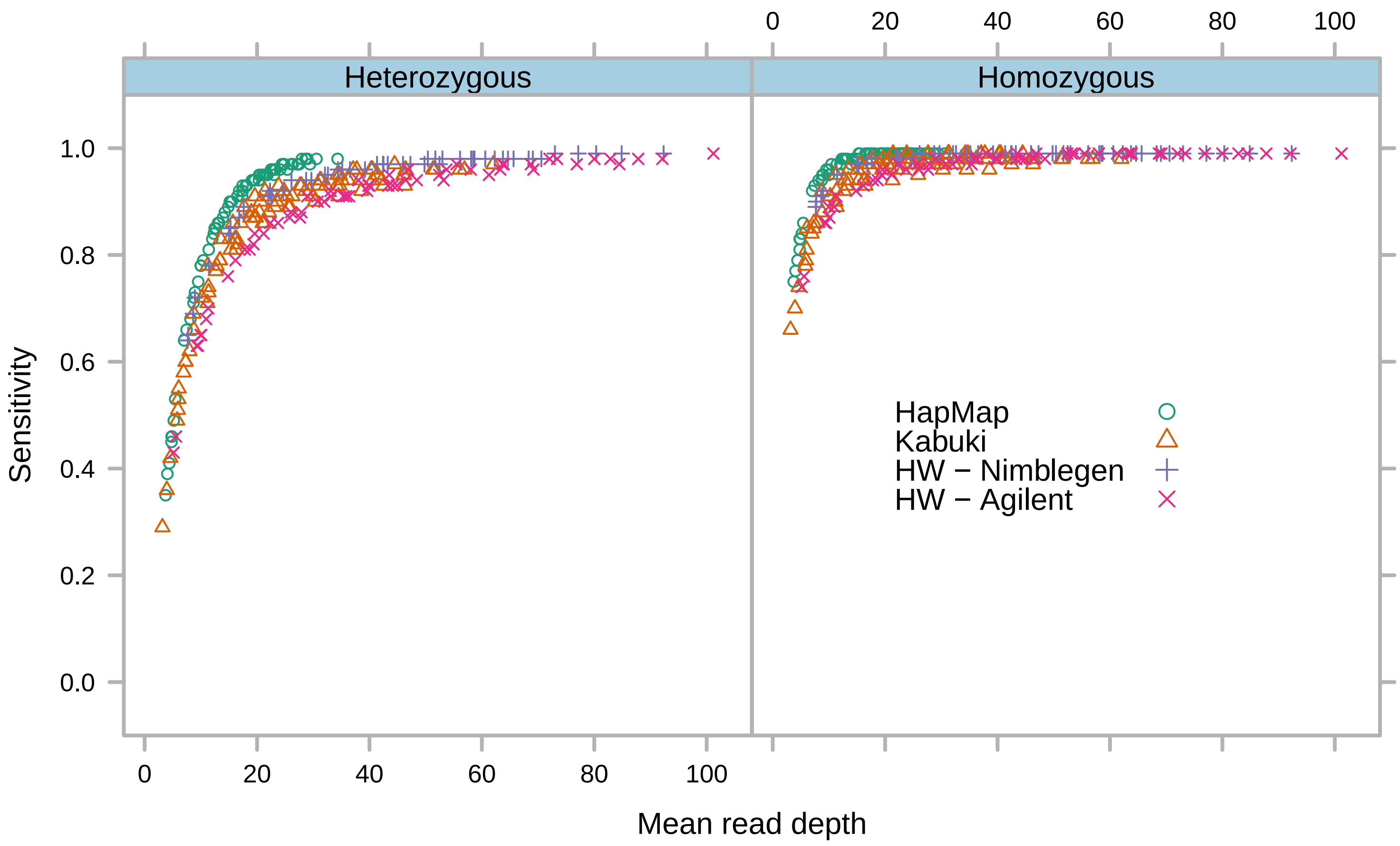

Supplementary Figure 4: Uniformity of read depth across targets from full CCDS target set. Mean read depth for non-overlapping tiles of length 100bp. All individual exomes from a given source show a similar distribution to the aggregate (data not shown). The x-axis has been truncated for display. The maximum values for the four distributions are: 142X, 3026X, 700X, and 4424X, with 0, 2085, 108, and 8941 tiles with mean depth over 400X, in order from left to right then top to bottom.

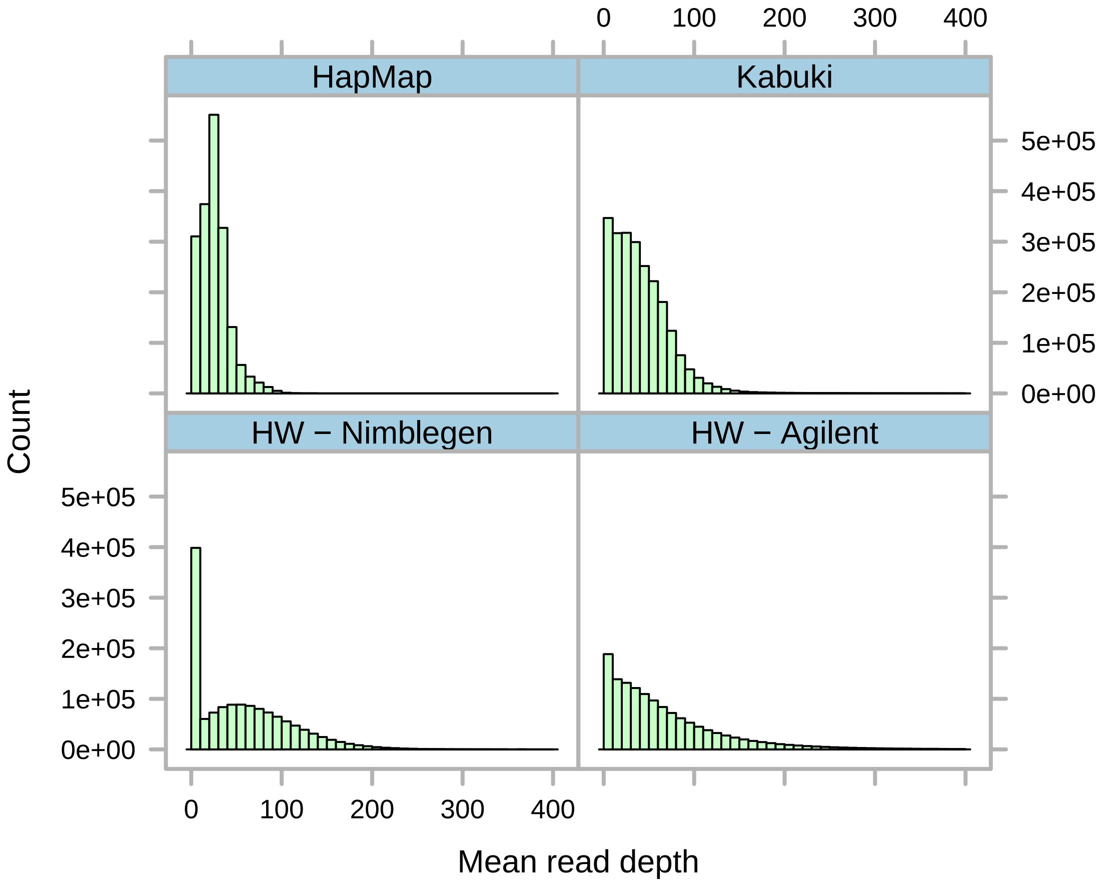

Supplementary Figure 5: Read depth in HW-Nimblegen sample for sites of novel heterozygous SNV calls from the corresponding HW-Agilent replicate.

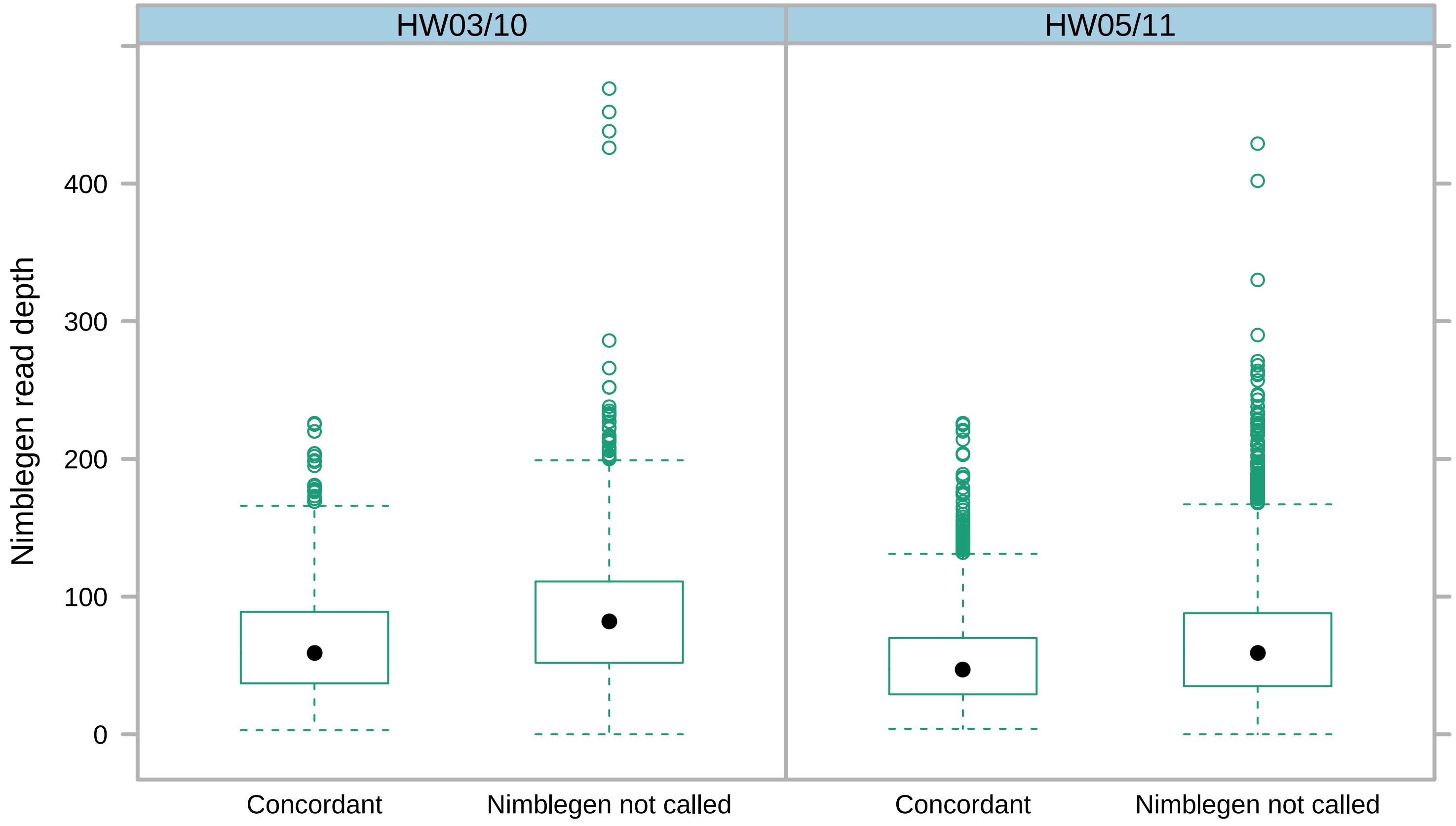

Supplementary Figure 6: G+C content distribution of 100bp target tiles classified as difficult or easy in at least one third of the exomes under study, compared with all other tiles ("Neither"). We considered a base well-covered if it had a read depth of at least 10, and a target region tile as well-covered if at least 90% of its bases were well-covered. "Difficult" target region tiles had no well-covered bases in the full alignments. "Easy" target regions were well-covered in the downsampled 0.1 alignments.

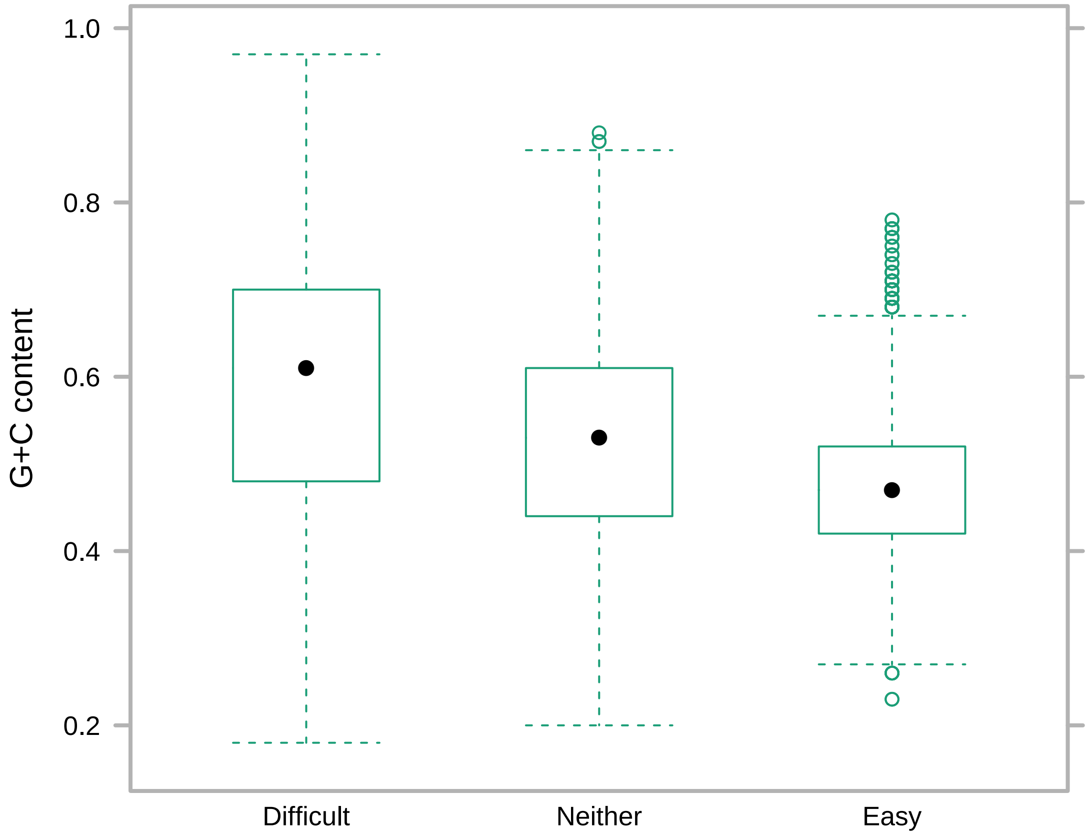

Supplementary Figure 7: Number of exomes sharing a difficult or easy target tile from full CCDS target, for non-overlapping tiles of length 100bp. The spike at 6 exomes in the difficult tiles histogram is primarily caused by tiles which were difficult only for the HW - Nimblegen set of exomes. Similarly, the spike at 14 exomes is caused by tiles which were difficult for both the HW - Nimblegen and the HapMap sets of exomes.

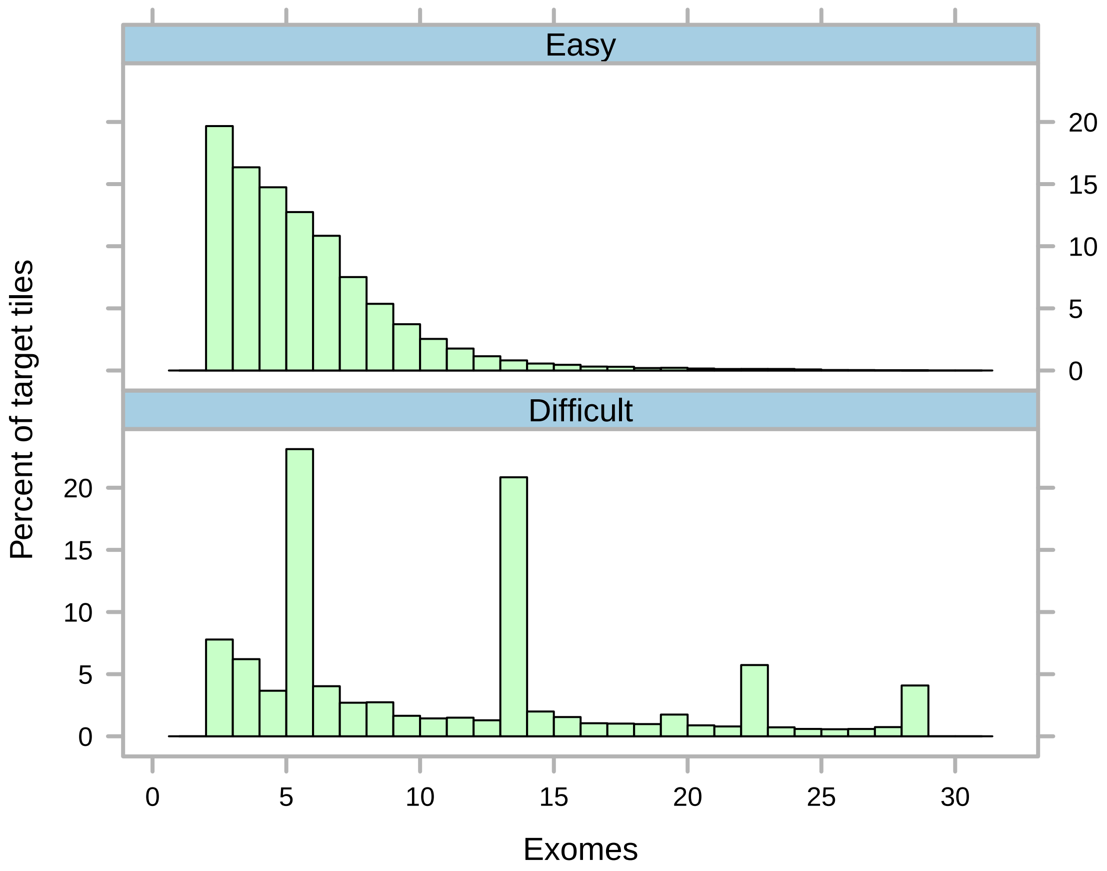

Supplementary Table 1: Sequencing and variant statistics for full alignments. The identification number in brackets for the Kabuki exomes is the kindred identification from Ng *et al.* (2010). Target is all exons from CCDS 20121025.

(a) Reads. Reads on target includes duplicate reads.

Source	Id	Unpaired reads	Read pairs	Unmapped reads	Duplication	Reads on target
HapMap	SRX005923 (NA12156)	96 794 096	0	42 376 281	0.38	28 535 286
	SRX005924 (NA12878)	112 713 195	0	73 850 868	0.38	45 944 812
	SRX005925 (NA18507)	102 411 337	0	64 206 533	0.38	43 565 656
	SRX005926 (NA18517)	104 243 170	0	45 558 312	0.39	39 199 920
	SRX005927 (NA18555)	95 868 427	0	51 540 046	0.39	36 176 546
	SRX005928 (NA18956)	104 942 582	0	50 890 268	0.39	43 758 021
	SRX005929 (NA19129)	105 718 468	0	53 649 298	0.37	43 595 172
	SRX005930 (NA19240)	105 851 627	0	57 118 784	0.36	46 481 789
Kabuki	SRS086451 (5)	55 554 344	0	8 922 948	0.26	25 337 330
	SRS086452 (10)	107 470 880	0	33 626 554	0.37	51 584 160
	SRS086453 (8)	69 910 410	0	11 271 662	0.40	32 279 661
	SRS086454 (1)	101 617 662	0	30 265 201	0.30	39 520 882
	SRS086455 (4)	28 420 086	21 977 080	52 444 732	0.20	26 979 319
	SRS086456 (2)	32 947 971	31 842 782	42 375 985	0.15	40 061 409
	SRS086457 (9)	34 649 387	29 112 346	40 984 344	0.16	35 573 753
	SRS086458 (7)	32 492 116	18 372 748	31 872 853	0.19	26 843 835
	SRS086459 (6)	33 076 820	25 383 251	41 771 754	0.16	35 567 936
	SRS086460 (3)	34 647 576	34 367 697	45 463 717	0.21	47 173 550
HW - Nimblegen	HW01	1 308 139	49 904 469	2 032 001	0.14	48 041 526
	HW02	1 897 449	84 216 154	3 155 080	0.23	75 217 183
	HW03	2 187 668	73 413 924	3 538 901	0.12	62 695 130
	HW04	2 515 907	72 956 766	4 123 779	0.21	61 690 997
	HW05	1 244 015	49 439 950	1 969 695	0.19	47 732 543
	HW06	2 272 384	85 484 712	3 967 312	0.43	73 853 952
HW - Agilent	HW07	1 805 307	53 068 827	2 225 554	0.15	49 867 900
	HW08	2 469 392	103 671 713	5 997 767	0.13	72 015 099
	HW09	2 218 302	88 423 465	4 469 173	0.13	70 108 401
	HW10	1 820 150	50 762 270	2 295 696	0.18	45 910 895
	HW11	1 952 722	93 109 004	2 537 229	0.29	82 536 576
	HW12	2 127 991	98 186 380	5 017 474	0.12	82 127 392

(b) Coverage.

Source	Id	Bases on target	Mean on-target read depth (X)	Granular 3rd quartile	Granular median	Granular 1st quartile	Bases over 15X
HapMap	SRX005923 (NA12156)	748 129 484	23.3	33	23	14	70.9
	SRX005924 (NA12878)	938 442 775	29.2	43	31	17	77.2
	SRX005925 (NA18507)	785 157 626	24.5	34	24	15	74.5
	SRX005926 (NA18517)	747 479 318	23.3	32	22	14	71.5
	SRX005927 (NA18555)	674 142 296	21.0	29	21	13	67.9
	SRX005928 (NA18956)	745 827 941	23.2	32	22	14	71.5
	SRX005929 (NA19129)	794 884 872	24.8	34	24	16	75.2
	SRX005930 (NA19240)	838 847 299	26.1	36	26	16	76.5
Kabuki	SRS086451 (5)	716 863 810	22.3	32	22	12	66.7
	SRS086452 (10)	1 336 500 389	41.6	62	42	21	82.2
	SRS086453 (8)	745 626 895	23.2	34	22	11	64.7
	SRS086454 (1)	1 211 593 918	37.7	55	37	19	79.4
	SRS086455 (4)	1 064 500 162	33.2	48	17	7	52.4
	SRS086456 (2)	1 688 303 250	52.6	73	48	26	85.5
	SRS086457 (9)	1 396 928 739	43.5	62	40	20	80.3
	SRS086458 (7)	1 057 429 708	32.9	47	31	16	76.1
	SRS086459 (6)	1 390 924 591	43.3	62	37	19	79.8
	SRS086460 (3)	1 868 207 905	58.2	80	52	28	86.2
HW - Nimblegen	HW01	1 673 052 868	52.1	82	47	10	72.4
	HW02	2 263 108 604	70.5	111	64	15	74.7
	HW03	1 968 695 632	61.3	99	58	13	73.7
	HW04	1 729 762 191	53.9	85	49	11	72.6
	HW05	1 562 492 306	48.7	75	43	10	72
	HW06	1 651 050 568	51.4	93	16	2	50.1
HW - Agilent	HW07	1 402 799 214	43.7	61	32	14	73.2
	HW08	2 539 615 761	79.1	104	57	27	85.6
	HW09	2 435 454 947	75.9	104	56	23	81.2
	HW10	1 253 477 679	39.1	55	29	12	69.5
	HW11	1 986 899 670	61.9	86	45	19	79.5
	HW12	2 922 944 682	91.1	122	68	32	86.3

(c) Variants in target regions.

			SNVs					
			HapMap3			Novel		
Source	Id	Indels	Heterozygous	Homozygous	Total	Heterozygous	Homozygous	Total
HapMap	SRX005923 (NA12156)	104	6 663	5 202	11 865	3 990	1 611	5 601
	SRX005924 (NA12878)	95	6 618	5 195	11 813	3 657	1 555	5 212
	SRX005925 (NA18507)	119	6 843	5 516	12 359	6 187	1 838	8 025
	SRX005926 (NA18517)	110	6 876	5 495	12 371	6 284	1 775	8 059
	SRX005927 (NA18555)	99	5 949	5 823	11 772	3 395	1 675	5 070
	SRX005928 (NA18956)	90	5 961	5 737	11 698	3 331	1 651	4 982
	SRX005929 (NA19129)	132	6 796	5 732	12 528	6 291	1 825	8 116
	SRX005930 (NA19240)	104	6 945	5 553	12 498	6 308	1 829	8 137
Kabuki	SRS086451 (5)	94	7 040	5 598	12 638	4 319	1 497	5 816
	SRS086452 (10)	128	7 328	5 691	13 019	4 993	1 740	6 733
	SRS086453 (8)	103	6 866	5 714	12 580	4 500	1 631	6 131
	SRS086454 (1)	135	7 526	5 627	13 153	5 082	1 768	6 850
	SRS086455 (4)	154	6 091	5 216	11 307	9 100	2 262	11 362
	SRS086456 (2)	159	7 303	5 693	12 996	5 220	1 934	7 154
	SRS086457 (9)	156	7 316	5 516	12 832	5 366	1 875	7 241
	SRS086458 (7)	156	7 116	5 868	12 984	5 025	1 981	7 006
	SRS086459 (6)	165	8 365	5 316	13 681	6 601	1 747	8 348
	SRS086460 (3)	172	7 262	5 642	12 904	5 260	1 939	7 199
HW - Nimblegen	HW01	135	6 169	4 838	11 007	4 250	1 518	5 768
	HW02	155	5 951	4 994	10 945	4 448	1 704	6 152
	HW03	140	5 843	4 902	10 745	3 627	1 697	5 324
	HW04	118	6 309	4 678	10 987	3 879	1 556	5 435
	HW05	135	6 400	4 789	11 189	4 396	1 679	6 075
	HW06	102	4 488	4 284	8 772	3 024	1 441	4 465
HW - Agilent	HW07	271	7 276	5 512	12 788	12 798	2 185	14 983
	HW08	226	7 551	5 756	13 307	5 331	1 927	7 258
	HW09	189	7 667	5 680	13 347	5 611	1 847	7 458
	HW10	235	6 598	5 781	12 379	12 597	2 107	14 704
	HW11	212	7 546	5 738	13 284	10 582	1 994	12 576
	HW12	184	7 899	5 751	13 650	6 434	2 041	8 475

Supplementary Table 2: Mean sensitivity at varying levels of read depth at a polymorphic site. Data shown in Figure 2. A complete table up to depth 100 is available in the file recall.tsv.

Depth	Heterozygous	Homozygous
0	0.00	0.00
1	0.00	0.06
2	0.00	0.89
3	0.30	0.97
4	0.52	0.99
5	0.67	0.99
6	0.76	0.99
7	0.81	0.99
8	0.84	0.99
9	0.88	0.99
10	0.90	0.99
11	0.92	0.99
12	0.94	0.99
13	0.96	0.99
14	0.97	0.99
15	0.97	0.99
16	0.98	0.99
17	0.98	0.99
18	0.98	0.99
19	0.99	1.00
20	0.98	1.00
25	0.99	1.00
30	0.99	1.00
35	1.00	1.00
40	0.99	1.00

Supplementary Table 3: Data sources and sample information.

Source	Samples	Reads	Capture	Sequencing
HapMap	8	single 76bp	2 custom Agilent 244K microarrays	Illumina GAII
Kabuki	10	single/paired 76bp	custom Agilent 1M aCGH array	Illumina GAII
HW - Nimblegen	6	paired 54bp	Roche NimbleGen 2.1M	Illumina GAII
HW - Agilent	6	paired 54bp	Agilent SureSelect All Exon 38M	Illumina GAII

Chang, H., Jackson, D. G., Kayne, P. S., Ross-Macdonald, P. B., Ryseck, R. P., and Siemers, N. O. (2011). Exome sequencing reveals comprehensive genomic alterations across eight cancer cell lines. *PLoS One*, **6**(6).

McKenna, A., Hanna, M., Banks, E., Sivachenko, A., Cibulskis, K., Kernytsky, A., Garimella, K., Altshuler, D., Gabriel, S., Daly, M., and DePristo, M. A. (2010). The genome analysis toolkit: a mapreduce framework for analyzing next-generation dna sequencing data. *Genome Res*, **20**(9), 1297–1303.

Ng, S. B., Bigham, A. W., Buckingham, K. J., Hannibal, M. C., McMillin, M. J., Gildersleeve, H. I., Beck, A. E., Tabor, H. K., Cooper, G. M., Mefford, H. C., Lee, C., Turner, E. H., Smith, J. D., Rieder, M. J., Yoshiura, K., Matsumoto, N., Ohta, T., Niikawa, N., Nickerson, D. A., Bamshad, M. J., and Shendure, J. (2010). Exome sequencing identifies MLL2 mutations as a cause of Kabuki syndrome. *Nat Genet*, **42**(9), 790–793.
